# Establishment of a reverse genetics system for an epidemic strain of porcine rotavirus JXAY01 type G5P[23]I12

**DOI:** 10.3389/fvets.2024.1512327

**Published:** 2025-02-12

**Authors:** Changjin Liu, Huangsiwu Wei, Xingyi Zhang, Wenjie Wu, Zhengqiao Shen, Feng Luo, Shunzhou Deng

**Affiliations:** ^1^Department of Preventive Veterinary Medicine, College of Animal Science and Technology, Jiangxi Agricultural University, Nanchang, China; ^2^The College of Life Science, Nanchang Normal University, Nanchang, China; ^3^Lushan Botanical Garden, Jiangxi Province and Chinese Academy of Science, Lushan, China; ^4^Jiangxi Jinyibo Biotechnology Company, Nanchang, China

**Keywords:** porcine rotavirus, reverse genetics, epidemic strain, monoreassortant, viral replication

## Abstract

Porcine rotavirus is one of the most important pathogens causing diarrhea in newborn piglets, and the genome of this virus contains 11 double-stranded RNA segments, which are easy to be recombined among strains to produce new strains with different antigenic properties. The reverse genetics system is an informative tool for studying virus biology. Recently, adaptable plasmid-based reverse genetics systems were developed for the porcine rotavirus OSU strain; however, such systems have not been developed for epidemic porcine rotavirus genotypes in China. In this study, we successfully established a reverse genetic system based on an epidemic strain of porcine rotavirus JXAY01 isolated in recent years, which was characterized by a specific genotype constellation: G5-P[23]-I12-R1-C1-M1-A8-N1-T7-E1-H1. 11 gene segments of porcine rotavirus JXAY01 were cloned into plasmid vectors similar to the SA11 system. JXAY01 genome segment plasmids were co-transfected with 10 complementary SA11 genome plasmids, and 11 monoreassortant strains were successfully rescued. Viral replication analyses of the parental SA11 strain and the monoreassortant strains showed that the structural protein replacement monoreassortants had reduced cell proliferation compared with the parental SA11 and non-structural protein replacement strains. The recombinant rJXAY01 strain could be successfully rescued using 11 pRG-JXAY01 plasmids. Whole genome sequencing showed 12 amino acid differences between the isolate JXAY01 and the recombinant rJXAY01, but there was no significant difference in their *in vitro* replication ability. This study reports the reverse genetic system, which lays the foundation for further understanding of porcine rotavirus molecular biology and novel vaccine development.

## 1 Introduction

Rotavirus infections often cause viral diarrhea in infants and animals, which can be transmitted between humans and animals and pose a serious threat to both human and animal husbandry (1). Porcine rotavirus primarily infects suckling piglets aged 1 to 4 weeks. The clinical symptoms include watery diarrhea, weight loss, and dehydration (2). Epidemiological studies show high antigenic positivity of porcine rotavirus in pig farms (3). Although rotavirus infection causes low diarrhea mortality and high morbidity in piglets, the loss of production caused by it poses a huge economic burden on pig farming.

Rotaviruses are a type of virus that have double-stranded RNA and are classified in the Rotavirus genus within the Sedoreoviridae family (4). Rotavirus comprises six structural proteins that come together to form non-enveloped viral particles with three layers. These particles contain a genome consisting of 11 segments. The RV genotypes are determined by two viral surface proteins: glycoprotein VP7, which is responsible for the G-type, and spike protein VP4, which determines the P-type (5). Porcine rotaviruses have been reported to consist of 12 G-type (G1 to G6, G8 to G12, and G26) and 16 P-type (P[1] to P[8], P[11], P[13], P[19], P[23], P[26] to P[27], P[32], and P[34]) viruses, and the combination of G-type and P-type viruses may take place randomly (1). The dual (G/P) tying method has been expanded into a comprehensive genome categorization system, which relies on nucleotide sequencing of all 11 RV segments and sets specific cut-off values for nucleotide percent identity for each segment. In this system, VP7-VP4-VP6-VP1-VP2-VP3-NSP1-NSP2-NSP3-NSP4-NSP5/6 RV genes are designated as Gx-P[x]-Ix-Rx-Cx-Mx-Ax-Nx-Tx-Ex-Hx (6). The RVA strains from China were the most prevalent genotypes for each segment: G9/G5/G4/G26- P13/P23/P7/P6- I5/I1- R1- C5- M1- A8- N1- T1/T7- E1- H1 (3, 7).

Reverse genetics is a powerful technology that allows for the creation of live viruses from cloned cDNA(s). It is an informative tool for studying various aspects of virus biology, such as viral replication, pathogenesis, and virus-host interactions (8). Additionally, it can be used to produce vaccine seed strains and viral vectors. RNA viruses with segmented genomes are more challenging to rescue because they require the simultaneous introduction of multiple genome-infectious clone molecules into a single cell. Until 2017, the first whole plasmid-based reverse genetic system for the monkey rotavirus strain SA11 was established (9). Rotavirus reverse genetics systems have been subsequently constructed for several species sources, including non-human primates (SA11 and RRV), humans (KU, CDC-9, Odelia, and RIX4414-like), cattle (UK, RF, BLR, and HM26), mice (rD6/2-2 g), birds (PO-13), and porcine (OSU) (10–20).

In this paper, 11 genomic cDNA plasmids were constructed using the isolated porcine rotavirus strain JXAY01 as a template. Successful rescue of 11 monoreassortant strains based on the SA11 reverse genetics system. The whole plasmid was successfully used to rescue the endemic porcine rotavirus strain rJXAY01.

## 2 Materials and methods

### 2.1 Cells, virus and plasmid

African green monkey kidney epithelial MA104 cells (ATCC CRL-2378) were cultured in Dulbecco’s modified Eagle’s medium (DMEM, Gibco) supplemented with 5% fetal bovine serum (FBS; Gibco). Baby hamster kidney cells stably expressing T7 RNA polymerase (BSR-T7/5 cells) were obtained from the Cell Resource Center, Peking Union Medical College (which is part of the National Science and Technology Infrastructure, the National Biomedical Cell-Line Resource, NSTI-BMCR, http://cellresource.cn). The cells were cultured in 1640 medium supplemented with 5% FBS. Epidemic porcine rotavirus strain JXAY01 isolated from Jiangxi Province, China, was propagated in MA104 cells grown in DMEM without serum and supplemented with 5 μg/mL trypsin (Solarbio, China). The plasmids used in the previously described reverse genetics systems (RGS) for the RVA strain SA11 (9) consisted of the 11 separate SA11 genome segment plasmids, together with three helper plasmids, pCAG-D1R, pCAG-D12L, and pCAG-FAST-p10 (kindly provided by Takeshi Kobayashi and received through Addgene).

### 2.2 Plasmid construction

The rescue vector plasmid pRG-Base (GenBank accession no. PQ736896), utilized in the reverse genetics system, was constructed by incorporating the HDV nuclease self-shearing sequence and the T7 terminator sequence into the restriction enzyme sites (SmaI and HindIII) of the pUC19 plasmid. The JXAY01 strain of porcine rotavirus was plaque-purified and passaged to the twentieth generation on MA104 cells. The whole-genome nucleotide sequences of the JXAY01 strain (GenBank accession no. PQ736897 to PQ736907) was determined using the illumina NovaSeq PE150 high throughput sequencing platform (Tsingke Biotech, Beijing, China). Primers for plasmid synthesis were prepared according to the instructions provided by the In-Fusion Cloning kit (Takara bio, China). The forward primers contain the T7 RNA polymerase promoter sequence and a sequence corresponding to the 5′ terminus of each viral segment. To construct the JXAY01 rescue plasmids pRG-JXAY01-VP1, pRG-JXAY01-VP2, pRG-JXAY01-VP3, pRG-JXAY01-VP4, pRG-JXAY01-VP6, pRG-JXAY01-VP7, pRG-JXAY01-NSP1, pRG-JXAY01-NSP2, pRG-JXAY01-NSP3, pRG-JXAY01-NSP4, and pRG-JXAY01-NSP5, each of which contains the full-length cDNA of the corresponding gene segment from strain JXAY01, full-length RV cDNAs were amplified by RT-PCR from viral dsRNA extracted from purified virions. The amplified cDNA segments were incorporated into pRG-Base by the process of In-fusion cloning. The viral cDNAs were flanked by T7 promoter and HDV ribozyme sequences in rescue plasmids. DNA sequencing was used to confirm each plasmid. The purification of all plasmids was carried out using the Endo Free Mini Plasmid Kit II (TIANGEN, China). The primer sequences utilized for plasmid synthesis can be provided by making a request.

### 2.3 Generation of recombinant rotaviruses

Recovery of SA11, monoreassortants, and rJXAY01 from cloned cDNAs was performed following previously published methods, with certain adjustments (9, 21). Briefly, a monolayer of BSR-T7/5 cells in 12-well plates was cultured for 24 h and then co-transfected with different combinations of plasmids using the TransIT-LT1 transfection reagent (Mirus Bio, United States). The following plasmid amounts were added to 125 μL Opti-MEM: Each of the 11 genome segment plasmids of either SA11 or PoRV-AY01 were added at an amount of 650 ng, except for the plasmids encoding NSP2 and NSP5, which were added at an amount of 1950 ng each (10). 650 ng of pCAG-D1R and pCAG-D12L, and 50 ng of pCAG-FAST. Following the addition of 17.5 μL of transfection reagent, the mixture was pre-incubated for 30 min at room temperature. Afterwards, BSR-T7/5 cells were rinsed three times with serum-free 1,640 media and then provided with 1 mL of this medium. Subsequently, the DNA mixture was introduced into the cells. After 48 h incubation at 37°C and 5% CO_2_, virus was harvested from the cells by three freeze/thaw cycles and then separated by centrifugation for 10 min at 3000 rpm. To passage, 500 μL of the liquid above the sediment was used to infect MA104 cells that were cultivated in 6-well plates. The supernatant containing the virus was treated with 15 μg/mL of trypsin for a duration of 1 h at a temperature of 37°C. Confluent MA104 cells cultivated in 6-well plates were rinsed twice with DMEM. The active virus was diluted in a 1:1 ratio with DMEM and introduced to the cells. Following a 1 h incubation at a temperature of 37°C and a CO_2_ concentration of 5%, the virus inoculum was removed. The cells were then washed once with DMEM before adding the maintenance medium, which contained 5 μg/mL of trypsin. The infected cells were harvested at 3 days post-infection or upon the detection of a visible cytopathic effect (CPE).

### 2.4 Plaque assay

Virus-containing cell lysates were activated as described above. Monolayers of MA104 cells in 6-well plates were rinsed with DMEM and exposed to 400 μL of virus that had been diluted 10-fold in a series. This exposure lasted for 1 h at a temperature of 37°C and a CO_2_ concentration of 5%. Following virus adsorption, the virus inoculum was removed. After washing the cells with DMEM, 2 mL/well overlay medium was added (1:1 ratio of 2.4% low melting point Agarose [sigma-aldrich, United States] and 2 × DMEM supplemented with) and incubated for 4 days. The cells were stained with 500 μL of Neutral Red Sterile Solution (Solarbio, China). After incubating for 3 h, the number of plaque and their respective diameters were determined using ImageJ software.

### 2.5 Growth kinetics of viruses

A monolayer culture of MA104 cells in 6-well plates was incubated overnight and subsequently infected with a virus at a multiplicity of infection (MOI) of 0.1 plaque-forming units (pfu) per cell. Following a 1 h absorption period at 37°C, the cells were rinsed twice with DMEM and subsequently placed in 2 mL of DMEM with 5 μg/mL trypsin at 37°C and 5% CO_2_. The cells were disrupted by the process of freeze/thawing after being incubated for 0, 12, 24, 36, and 48 h post infection. The viral titre of each sample was measured using plaque assays.

### 2.6 Electrophoresis of dsRNA

Viral dsRNA was purified from lysates of virus-infected cells using the RNA Viral Genome Extraction Kit (Solarbio, China), according to the manufacturer’s instructions. Genomic RNAs were fractionated on 10% polyacrylamide gel with a Tris-glycine buffer. Electrophoresis was conducted at room temperature for 240 min at a constant current of 130 V. polyacrylamide gels were visualized by silver nitrate staining.

### 2.7 Immunofluorescence analysis

Monolayer MA104 cells in 96-well plates were infected for 24 h with Lysed supernatant from BSR-T7/5 cells 48 h post-transfection activated with trypsin. Then fixed with 10% paraformaldehyde. The cells were incubated for 1 h at 37°C with anti-A group rotavirus VP6 protein monoclonal antibody 4G7 diluted at 1:2000. Monoclonal antibody 4G7 was prepared in our laboratory using a porcine rotavirus isolate as the immunogen and has been confirmed to target the VP6 protein of group A rotavirus of different genotypes, including I1, I5, and I12. After, the cells were washed 3 times with PBS and then incubated for 1 h at 37°C with FITC-labelled goat anti-mouse monoclonal antibody (diluted 1:500, Solarbio, China), Images were acquired under fluorescence microscope.

### 2.8 Sequence analyses

Extraction of viral RNA from centrifuged supernatants of infected cell lysates. The whole-genome nucleotide sequences of the JXAY01 and rJXAY01 strain was determined using the illumina NovaSeq PE150 high throughput sequencing platform (Tsingke Biotech, China). Data were analyzed with Geneious Prime (Version 2023.0.4, Biomatters).

### 2.9 Statistical analysis

Graphs were generated by GraphPad Prism 9.0.0. The data is presented as the mean as well as the standard deviation, derived from three separate trials, with each experiment including technical duplicates, unless specified otherwise. *p* values were calculated using the ratio paired *t* test. A significance level of <0.05 was regarded statistically significant with *p* ≤ 0.05 (*), *p* < 0.01 (**), and *p* < 0.001 (***).

## 3 Results

### 3.1 Porcine rotavirus JXAY01 monoreassortants viruses were generated based on simian RV SA11 strains

**Figure 1:**
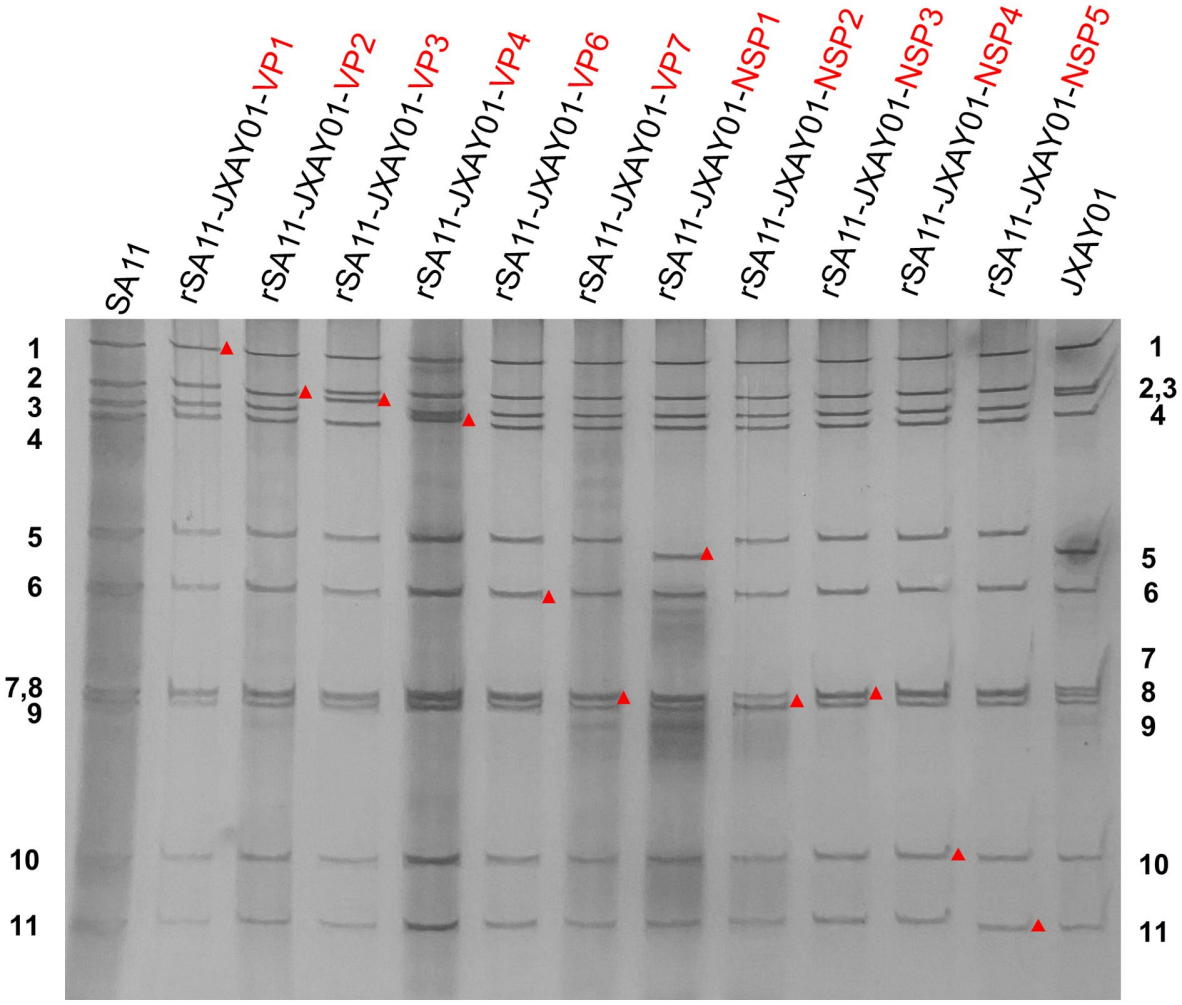
PAGE of viral genomic dsRNA extracted from SA11, native JXAY01 and monoreassortants. The numbers on the side indicate the order of the genomic dsRNA segments of SA11 and JXAY01. The red markers indicate recombinant segments.

In order to create a reverse genetics system for PoRV, we produced cloned cDNA that encodes each of the 11 gene segments obtained from strain JXAY01(G5P[23]I12). cDNAs encoding the 11 JXAY01 dsRNA gene segments were inserted into plasmids at specific locations that were surrounded by the T7 promoter and Hepatitis delta virus ribozyme sequences. Infectious cloning plasmids were required to contain the exact viral genome ends, and we sequenced the plasmids and verified the accuracy of each plasmid based on the SA11 reverse genetics system. A collection of genetically engineered monoreassortants has been created, consisting of 10 segments derived from SA11 and 1 segment from JXAY01. In PAGE verification, the viral dsRNAs obtained from the 11 rescued monoreassortants moved to the identical locations as the corresponding segments of JXAY01 with a SA11 backbone (Figure 1). The gene segments derived from strain JXAY01, when arranged on the genetic background of strain SA11, are indicated by red markers. The results demonstrated that all 11 infectious clones of porcine rotavirus, pRG-JXAY01, were accurately produced and are suitable for strain rescue.

### 3.2 Size of plaque in parental and monoreassortants rSA11-JXAY01

**Figure 2:**
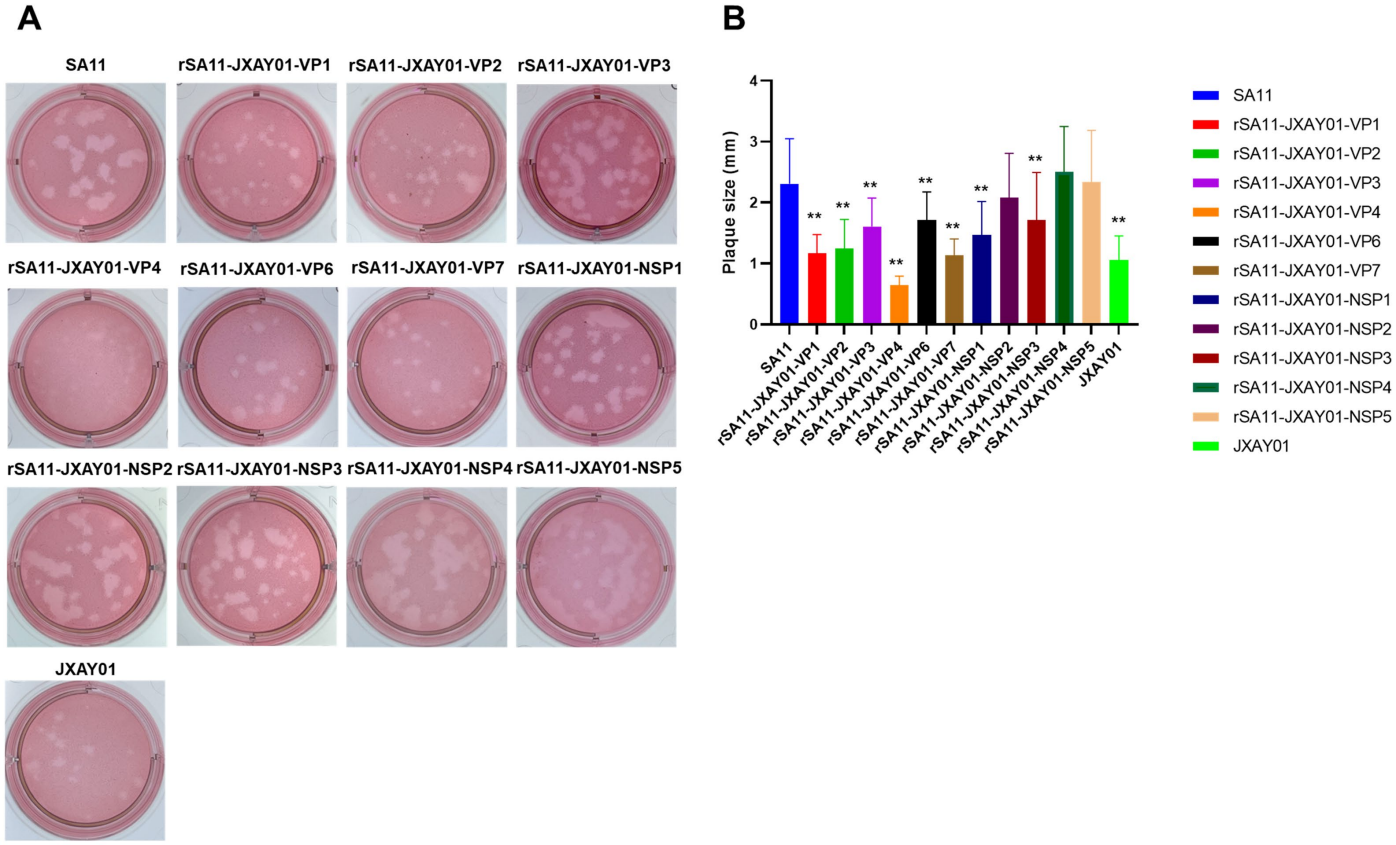
Plaque size comparison. **(A)** Plaques were formed on MA104 monolayers and identified at 4 days post-infection using Neutral Red staining. Displayed are representative images of viral plaques. **(B)** The dimensions of a minimum of 30 plaques, picked randomly from two separate plaque tests, were quantified using the GraphPad Prism software and expressed as area in relative units. The mean values and standard deviation are displayed. The statistical significance was assessed using a Student’s t-test. The asterisks are used to highlight significant differences (*p ≤ 0.05; **p ≤ 0.01; ***p ≤ 0.001).

The size of plaque formation was evaluated on MA104 cells infected with the SA11 strain, the monoreassortants rSA11-JXAY01, and the JXAY01 strain after a duration of four days. Plaque formation assays were performed on 13 strains, comprising 11 single-segment recombinant SA11 strains and two parental strains, SA11 and JXAY01. The plaque size exhibited significant variation among certain strains in comparison to the original strains (Figure 2A). For the statistical analysis, 30 plaques were randomly selected from two distinct replications. In comparison to the SA11 strain, the rSA11-JXAY01-VP1, rSA11-JXAY01-VP2, rSA11-JXAY01-VP3, rSA11-JXAY01-VP4, rSA11-JXAY01-VP6, rSA11-JXAY01-VP7, rSA11-JXAY01-NSP1, and rSA11-JXAY01-NSP3 demonstrated reduced plaque size, with statistically significant differences (*p* < 0.01). The rSA11-JXAY01-NSP2, rSA11-JXAY01-NSP4, and rSA11-JXAY01-NSP5 viruses exhibited decreased plaque size. No obvious disparity existed in the measurements of the plaque when compared to SA11 (Figure 2B).

### 3.3 Growth curve of parental and monoreassortants rSA11-JXAY01

**Figure 3:**
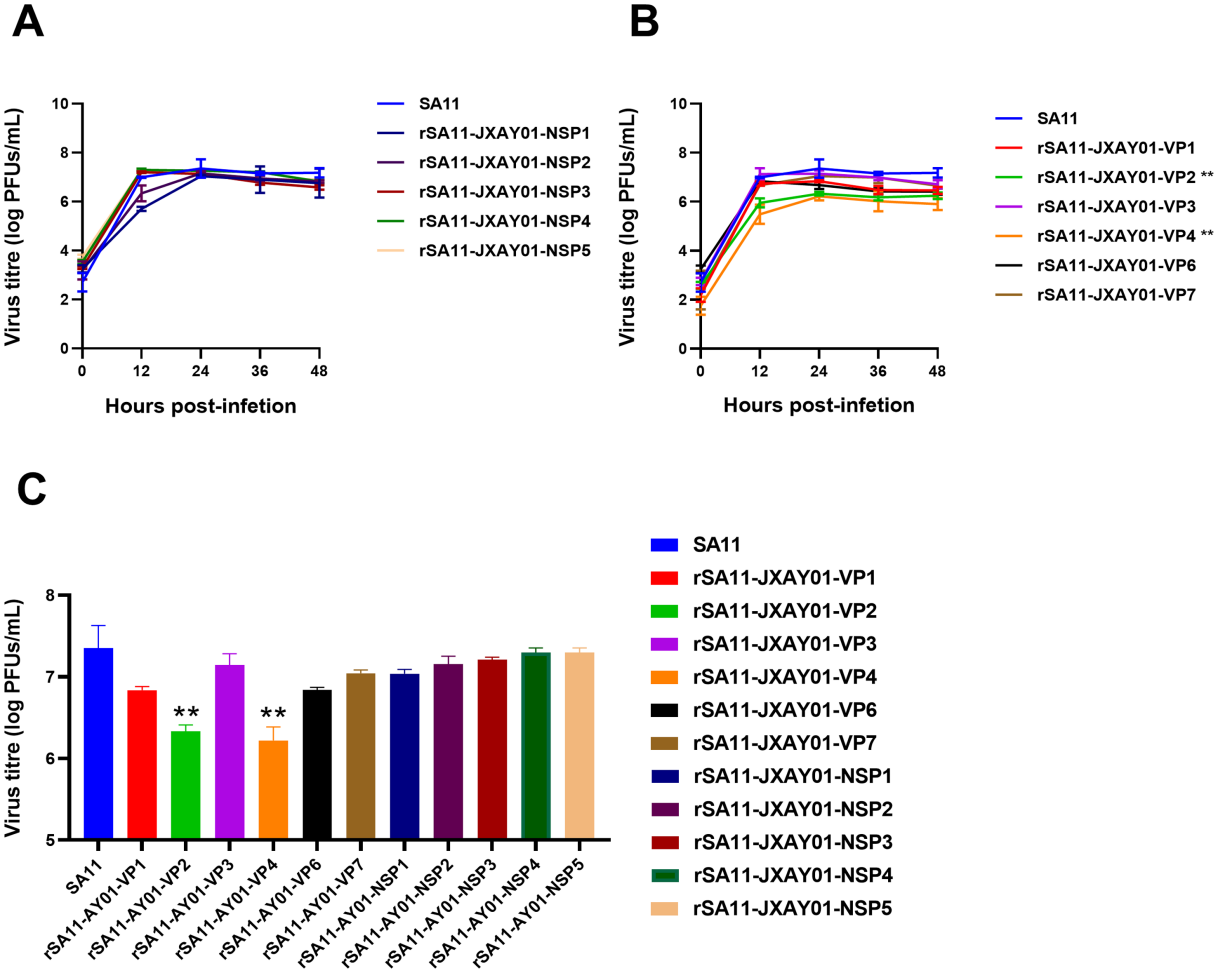
Growth kinetics of reassortant viruses. **(A,B)** Confluent MA104 cells cultured in 6 wells were exposed to an infection with a multiplicity of infection (MOI) of 0.1. At the specified time intervals, samples were collected and the number of plaque-forming units (PFUs) was calculated using plaque assay. **(C)** The illustrated data are the means obtained from three separate experiments, and the error bars indicate the standard deviation. The viral titers at 24 h post-infection were used for statistical analysis, and the results showed that the viral titers of the VP2 and VP4 single-segment recombinant strains were significantly reduced (*p ≤ 0.05; **p ≤ 0.01; ***p ≤ 0.001).

The parental strain SA11 and 11 single segment recombinant rotavirus rSA11-JXAY01 were infected with MA104 at a multiplicity of infection (MOI) of 0.1. The growth curves and peak titre of the infected cells were then assessed. According to Figure 3, the recombinant rSA11-JXAY01 strain, in which the non-structural protein (NSP) of JXAY01 was replaced, showed no significant differences compared to the parental SA11 strain in terms of growth curves and peak viral titre (Figure 3A). Among the strains where the structural protein (VP) was replaced, rSA11-JXAY01-VP2 and rSA11-JXAY01-VP4 exhibited a noteworthy decrease in viral multiplication when compared to the original SA11 strain (Figure 3B). The ability of the rSA11-JXAY01-VP2 and rSA11-JXAY01-VP4 viruses to reproduce in the structural protein (VP) replacement strain was significantly less than that of the original SA11 strain (*p* < 0.01, Figure 3C).

### 3.4 Porcine rotavirus strain JXAY01 whole plasmid rescue

**Figure 4:**
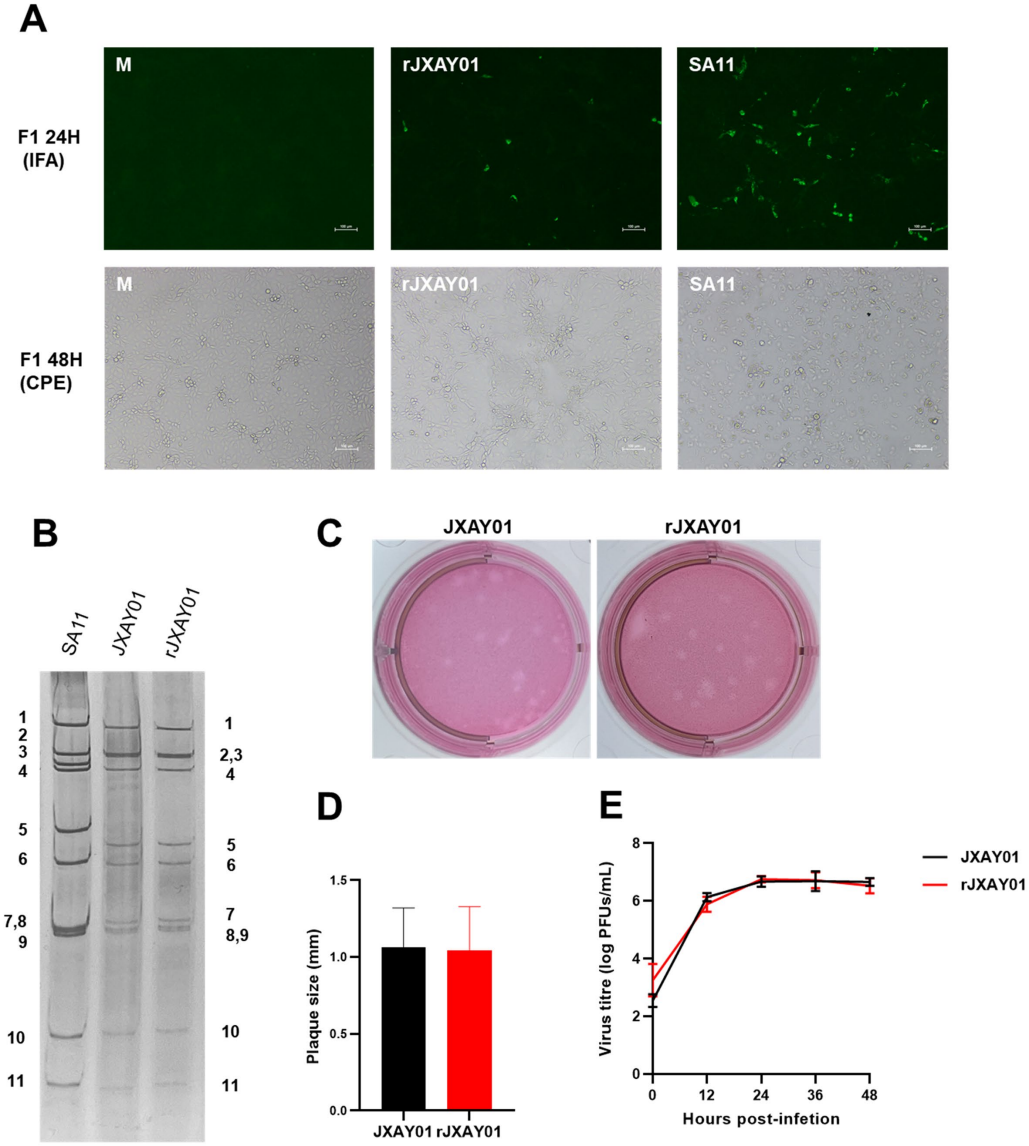
Establishment of a reverse genetics system for Porcine rotavirus strain JXAY01. **(A)** Immunofluorescence detection and CPE observation of the whole plasmid rescue strain rJXAY01. **(B)** Electrophoretic patterns of dsRNA from SA11, native JXAY01 and rJXAY01. **(C)** Plaque morphologies of native JXAY01 and rJXAY01 on MA104 cells were detected by neutral red staining and sizes. **(D)** The plaque size were measured using ImageJ software. Statistical analysis shows no significant difference in the plaque sizes between the two strains. **(E)** Growth kinetics of native JXAY01 and recombinant strain rJXAY01 in MA104 cells. Statistical analysis shows no significant difference in the viral titers between the two strains (*p ≤ 0.05; **p ≤ 0.01; ***p ≤ 0.001).

A total of eleven pRG-JXAY01 infectious cloned plasmids were utilized, which were confirmed through sequence determination and single segment group recombination. These plasmids were employed for the complete plasmid rescue of pig rotavirus rJXAY01. For the entire plasmid rescue of porcine rotavirus rJXAY01, we utilized 11 pRG-JXAY01 infectious clone plasmids that were confirmed using sequence determination and segment group recombination. After 48 h of transfection, the BSR-T7/5 cells in the transfected wells were subjected to freeze-thawing and collected as the F0 viral solution. The MA104 cells in the 96-well plates were then infected with the activated F0 viral solution, diluted at a ratio of 1:10. After 24 h, an immunofluorescence assay (IFA) was performed. MA104 cells were infected with a 1:1 dilution of the activated F0 virus solution in 6-well plates. This was done to continuously observe the cytopathic effect (CPE). Figure 4A demonstrates that both the rescue strain rJXAY01 and the positive rescue control SA11 exhibited specific fluorescence 24 h after infection. The rJXAY01’s rescue success rate (4/5) may be marginally inferior to that of the SA11 (5/5) due to the characteristics of viral replication. The fluorescence intensity of rJXAY01 was inferior to that of SA11. The fluorescence emitted by the rJXAY01 was detected in the wells that underwent amplification 48 h after infection. After 48 h of infection, only a modest quantity of cytopathic effect (CPE) was found in rJXAY01. In contrast, the SA11 cells in the infected wells exhibited full illness, with over 80% of the cells becoming detached and lysed. The cells in the infected wells of the SA11 strain exhibited complete disease symptoms, with over 80% of them becoming detached. The 1:1 F1 viral solution was utilized for subsequent passaging, and the rescue strain rJXAY01 induced full lesion and lysis of MA104 cells 24 h after infection. At 24 h after infection, the MA104 cells were fully damaged and separated. The virus obtained from this process was then collected for the purpose of purifying and identifying it.

The nucleic acid was isolated from the genome of the porcine rotavirus rescue strain rJXAY01 and analyzed using nucleic acid electrophoresis (Figure 4B). The nucleic acid electrophoresis results revealed that the migrating bands of the rescue strain were indistinguishable from those of the isolate JXAY01, while the bands of the rotavirus SA11 exhibited dissimilarities. The proliferation properties of the JXAY01 isolate and the rJXAY01 rescue strain on MA104 cells were assessed. There was no notable disparity in the size of plaque morphologies generated on MA104 cells between the rescue strain and the isolate (Figures 4C,D). The viruses were administered with a MOI of 0.1. The MA104 cells were infected with the virus at a MOI of 0.1. The viral growth curves were then plotted, revealing no notable disparity in the viral titre and proliferation process between the rescue strain and the isolate (Figure 4E). The findings indicated that there was no notable disparity in the viral titre and proliferation process between the rescue strain and the isolate.

### 3.5 Sequence analysis of rescue and parental strains

The rescued strain rJXAY01 was plaque-purified and subsequently propagated to the fifth generation for sequencing. The complete sequences have been deposited in GenBank and assigned accession numbers as follows: PP975110 ~ PP975120. A comparative analysis was conducted between the complete genome sequencing of the rescued strain rJXAY01 and the parental isolate JXAY01. Table 1 reveals that there were a total of 20 variations in nucleotides and 12 variations in amino acids between the two strains. Specifically, there were 5 nucleotide mutations in the NSP1 gene, leading to 3 amino acid alterations, as well as 2 amino acid changes in VP4 and 1 amino acid change in VP7. Additionally, VP4 and VP7 underwent 2 and 1 amino acid alterations, respectively. The mutations listed did not result in any notable variation in the proliferation properties of the rescue. Furthermore, the fact that the strains were successfully saved indirectly demonstrates that these mutations did not hinder the rescue process.

**Table 1:** Nucleotide and amino acid changes in parental and rescue strains.

Gene	Nucleotide difference	Amino acid exchange
VP1	NC	NC
VP2	NC	NC
VP3	T770C/A1702G	I551M
VP4	A432G/T1227C/C1424T	I141M/S472L
VP6	C126T	NC
VP7	A393G/G956T	W289L
NSP1	A48G/A292G/G580A/A960G/A1386T	D13G/N317S/Y459F
NSP2	NC	NC
NSP3	T388C/A606G/T1058C	M106T/K179E
NSP4	A248G/G480A/T767C	I54M/D132N
NSP5	G468A	R148K

## 4 Discussion

The plasmid-based rotavirus reverse genetics technology has proved crucial in both fundamental and practical research (22, 23). The establishment of a reverse genetic system for rotavirus strains from various sources and hosts has significantly expanded the scope of research on rotavirus host specificity, viral replication gene regulation, heterotypic immunoprotection, and the development of new vaccines (21, 24, 25). Rotaviruses are commonly spread between different locations, and the ongoing task of discovering and categorising strains worldwide is always evolving. Nevertheless, the reverse genetic approaches employed for the investigation of rotavirus strains generally rely on transmissible strains that have been acclimated to laboratory cells and were obtained and cultivated in previous years (13–15). The sole existing porcine rotavirus complete plasmid reverse genetics method presently relies on the OSU strain, which was obtained by the Ohio State University in 1975, and differs significantly from the generally prevalent strains observed in recent times (17). In this study, we successfully established a reverse genetic system based on a porcine rotavirus strain JXAY01 that was isolated from China in 2021. The genomic constellation of this strain was G5-P[23]-I12-R1-C1-M1-A8-N1-T7-E1-H1. Epidemiological surveys indicate that in China, the prevalence of P[23] rotavirus is ranked second, following P[13] rotavirus (3, 26). Rotavirus type I12 is infrequently observed and commonly linked to transmission between humans and pigs (27, 28).

The untranslated regions (UTRs) located at the ends of rotavirus segments exhibit variations in length and consist of sequences and structural features that play a role in the regulation of transcription and packaging of viral genome (29, 30). The 5’UTRs of eight genome segments (gs3, gs5-6, gs7-11) from the simian SA11 strain exhibited a potent inhibitory effect on the production of viral proteins in uninfected cells when using T7 polymerase-driven cDNAs expression (29). The final four GACC nucleotides of viral mRNA are crucial for optimal translation, and both the NSP3 eIF4G- and RNA-binding domains are necessary (30). In this study, the plasmid was constructed by designing primers for conserved sequences at the end of the high-consistency sequence. The UTRs of the pRG-JXAY01 plasmid showed a strong similarity to the human rotavirus Odelia and KU strains, which have been successfully rescued from virulent strains. However, it exhibited significant differences from the monkey rotavirus SA11 strain, the bovine rotavirus RF strain, and the avian rotavirus PO-13 strain.

This study aimed to rescue porcine rotaviruses by utilising the similar reverse genetic system of monkey rotavirus strain SA11 (9). Initially, we successfully recovered 11 monoreassortant rotavirus rSA11-JXAY01 strains. Segmented RNA viruses are susceptible to gene segment exchange to form new viruses during co-infection occurrences. The successful rescue of the 11 recombinant strains indicated that there were no genetic restricted on the recombination of porcine rotavirus with SA11. The rescue and proliferation features of rotavirus recombinant strains were regulated by strain-specific interactions between rotavirus capsid proteins (31).

All replacements of structural proteins (VP) and non-structural protein (NSP) substitutions in the recombinant strains with a single segment were successful. The text mentions substitutions in the VP and NSP of the rSA11-JXAY01 virus, namely in the NSP1 and NSP3 proteins. The formation of viral plaques was significantly diminished in rSA11-JXAY01-NSP1, rSA11-JXAY01-NSP2, and rSA11-JXAY01-NSP3 as compared to the original SA11 strain. The three remaining recombinant strains exhibited no notable variations in plaque formation compared to SA11.

VP4 is a key component of rotavirus susceptibility to infection and rotavirus rescue. VP4 is the key to rotavirus infectivity and rotavirus rescue, and substitution of VP4 from strains with poor *in vitro* proliferative capacity could not rescue the single-segment recombinant rSA11 strain. The single segment recombinant strain rSA11-VP4 could not be rescued by replacing VP4 with that of a less proliferative strain in vitro (32). After replacing the KU-VP4 segment with the SA11-VP4 segment, the single-segment recombinant rKU-VP4SA11 was successfully rescued, and the proliferative capacity of this strain was significantly higher than that of the parental KU strain and similar to that of SA11 (16). The 11 single-segment recombinant rSA11-JXAY01 strains rescued from this study were subjected to etch size and growth curve measurements. The dimensions and rate of expansion of the 11 individual recombinant rSA11-JXAY01 strains recovered in this investigation were measured. The viral titre of rSA11-JXAY01-VP4 was reduced compared to the original strain, and it caused an etch on MA104. The rSA11-JXAY01-VP4 exhibited a reduced viral concentration compared to the original strain and produced the least lesion on MA104.

The rJXAY01 strain was successfully rescued using the validated 11 infectious clones of plasmid pRG-JXAY01. There was no significant difference between the rescued strain and the isolate JXAY01 in terms of etch formation and viral growth curve. High-throughput sequencing was performed on the rescued strain to compare its performance with that of JXAY01. High-throughput sequencing was performed to compare the nucleotide and amino acid sequence differences between the rescued strain and the parental strain, and the results showed that there were 20 nucleotides and 12 amino acid sequences in the rescued strain compared with the parental strain. Rotaviruses often undergo mutations during *in vitro* and ex vivo generations (33, 34) and some cell-adapted mutations enhance the proliferation of the virus in the corresponding cell lines (35). The mutation probability of rotavirus probability of mutation is influenced by cell lineage and can affect virus virulence (36). During the establishment of the reverse genetic system in this study, the virus transmission, plasmid construction, and virus rescue may have caused mutations during the establishment of the reverse genetic system in this study. There was no significant difference between the rescued strain and the parental strain at the cell proliferation level, and the existence of viral virulence alteration should be further investigated.

In conclusion, this experiment successfully established a reverse genetic system based on the porcine rotavirus isolate JXAY01. It lays the foundation for the study of host specificity, virulence genes and virulence mechanism of porcine rotavirus disease at the molecular level, and provides a basis for the development of new porcine rotavirus strains. It also provides a tool for the development of new porcine rotavirus vaccines.

## Data Availability

The data presented in the study are deposited in GenBank (https://www.ncbi.nlm.nih.gov/genbank/), The plasmid pRG-Base (GenBank accession no. PQ736896), porcine rotavirus JXAY01 strain (GenBank accession no. PQ736897 to PQ736907) and rescued porcine rotavirus rJXAY01 strain (GenBank accession no. PP975110 to PP975120).
